# Universal health coverage: a commitment to essential surgical, obstetric, and anesthesia care, World Health Summit 2021 (PD 20)

**DOI:** 10.1186/s12919-023-00258-x

**Published:** 2023-07-12

**Authors:** Manon Pigeolet, Selam Degu, Isabella Faria, Matthew T. Hey, Tayana Jean-Pierre, Don E. Lucerno-Prisno, Ali Jafarian, Natalia Kanem, John G. Meara, Lia Tadesse Gebremedhin, Cherian Varghese, Tarsicio Uribe-Leitz, Kee B. Park

**Affiliations:** 1 The Program in Global Surgery and Social Change, Harvard Medical School, Boston, MA USA; 2 Faculty of Medicine, Université Libre de Bruxelles, Brussels, Belgium; 3 Department of Pediatric Orthopedics, Necker University Hospital, Université Paris Cité, Paris, France; 4 Department of General Surgery, Imam Khomeini Hospital Complex, Tehran, Iran; 5 United Nations Population Fund (UNFPA), Headquarters, New York City, NY USA; 6 Federal Ministry of Health, Addis Ababa, Ethiopia; 7 Department of Healthier Populations & NCDs, WHO South East Asia Regional Office, New Delhi, India

## Introduction

Although increasingly recognized as an indivisible, indispensable part of universal health coverage (UHC), limited progress has been made in recent years to advance surgical, obstetric and anaesthesia care around the world. Over five billion people remain without access to safe surgical care.

Strong health systems are key in dealing with health crises, as was illustrated by the recent COVID-19 pandemic, in which the surgical ecosystem was integral to the pandemic response. Surgical systems have a direct relation with the four pillars of UHC: (1) the availability of services, (2) resources and equipment, (3) demand, and (4) access to services of health. The weakening of these four components impacts the provision and utilization of surgical care, which leads to a reduction in the coverage of surgical health services with an increase in deaths.

An integrated and holistic approach to build up health systems that includes increased emphasis on surgical care delivery is currently lacking. It is important to bring all key stakeholders—from academia, the private sector, government organizations, philanthropy and non-governmental organizations, and others—to the table to define the innovative solutions and pave the way forward.

From October 24–26, 2021, a group of leaders from politics, science and medicine, the private sector, and civil society convened for the 2021 World Health Summit. The panel discussion *UHC: A Commitment to Essential Surgical, Obstetric and Anesthesia Care* (PD 20) [1] was held on October 25, 2021 and included five framing talks followed by a discussion. The panel was moderated by workshop co-chairs Dr Kee B. Park, Director of Policy and Advocacy at Harvard Medical School’s Program in Global Surgery and Social Change, USA, and Dr Tarsicio Uribe-Leitz, affiliate Faculty of Harvard Medical School’s (HMS) Program in Global Surgery and Social Change, instructor in surgery at HSM, Investigator/Data Manager at Brigham and Women’s Hospital Center for Surgery and Public Health, USA. Featured speakers were:

- Prof Dr Ali Jafarian, professor and former chancellor at Tehran University of Medical Sciences, Iran
- Dr Natalia Kanem, United Nations Under-Secretary-General, Executive Director of the United Nations Population Fund (UNFPA), USA
- Prof Dr John G. Meara, Steven C. and Carmella R. Kletjian Professor of Global Health and Social Medicine in the field of Global Surgery at Harvard Medical School and plastic surgeon-in-chief at Boston Children’s Hospital, USA
- Dr Lia Tadesse Gebremedhin, Minister of Health at the Federal Ministry of Health, Ethiopia
- Dr Cherian Varghese, Cross cutting lead, NCD and special initiatives, Department of Noncommunicable Diseases at the World Health Organization, Switzerland

Park opened the session by highlighting that more than half of the world’s population lacks timely access to safe and affordable surgical care. In the absence of surgical services, unrepaired fractures can lead to permanent disabilities, mothers experiencing complications in childbirth may die or develop fistulas, and cancers might not be diagnosed until resection is no longer an option. This panel was designed to spotlight the indispensable and indivisible nature of surgical, obstetric, and anaesthesia (SOA) care and its importance in achieving UHC.

## Global surgery in turbulent times: from UHC and SDGs to pandemic preparedness

Dr John G. Meara, Steven C. and Carmella R. Kletjian Professor of Global Health and Social Medicine in the field of Global Surgery at Harvard Medical School and plastic surgeon-in-chief at Boston Children’s Hospital, US, provided a brief historical background of the evolution of global surgery in the past 40 years. Dr Halfdan Mahler, who served as World Health Organization (WHO) Director-General from 1973 to 1988, was among the first global leaders to link surgery and social inequity. In a 1980 address, he stated that “the vast majority of the world’s population has no access whatsoever to skilled surgical care.” [2] Later in his speech, Mahler linked access to surgery with social equity and justice, saying, “I beg of you to give serious consideration to this most serious manifestation of social inequity in health care.” In 2008, Paul Farmer and Jim Kim published an article that described global surgery as “the neglected stepchild of global health.” [3] This vivid metaphor galvanized the global surgery community. In 2015, The World Bank published a volume on global surgery [4] and the Lancet Commission on Global Surgery (LCoGS) published its findings [5]. Meara emphasized that World Health Assembly Resolution 68.15, passed in 2015, contributed to this momentum by codifying the strengthening of emergency and essential surgical and anaesthesia care as a component of UHC [6].

### The Lancet Commission on Global Surgery

Meara outlined four outputs of the report of the LCoGS: a vision, key messages, indicators, and the concept of the national surgical, obstetric, and anaesthesia plan (NSOAP). The LCoGS declared a vision for global surgery: universal access to safe, affordable surgical and anaesthesia care when needed. In reviewing the five key messages established by the LCoGS (see Table 1), Meara emphasized that financing surgical care is not merely an expenditure, it is an investment; US$350 billion in funding for low- and middle-income countries (LMIC) over a 15-year period could add as much as US$12 trillion to those economies. He highlighted that surgery is not a vertical intervention or separate entity, but rather a service that is integrated throughout health care and therefore should be included in national health programs.

**Table 1:** Key messages from the Lancet Commission on Global Surgery [5]

In 2015, the LCoGS developed five key messages about the status of surgical care worldwide
5 billion people lack access to safe, affordable surgical and anaesthesia care when needed
143 million additional surgical procedures are needed each year to save lives and prevent disability
81 million individuals face catastrophic health expenditure seeking surgery and anaesthesia each year
Investment in surgical and anaesthesia care is affordable, saves lives and promotes economic growth. An
investment of $350 billion would contribute an estimated $12 trillion in economic growth
Surgery is an indivisible, indispensable part of health care

As tasked by Richard Horton, editor-in-chief at *The Lancet*, The Commission identified six indicators for tracking progress in the surgical ecosystem [5]:

1. 2-h access to surgery: the percentage of the population within two hours of a hospital that can provide safe surgery
2. SOA surgical workforce, measured per 100,000 population
3. Surgical volume at the national level
4. Perioperative mortality rate: all-cause death prior to patient discharge
5. Impoverishing expenditure: protection against impoverishing expenditures for patients and families
6. Catastrophic expenditure: protection against catastrophic expenditures for patients and families

**Fig. 1:**
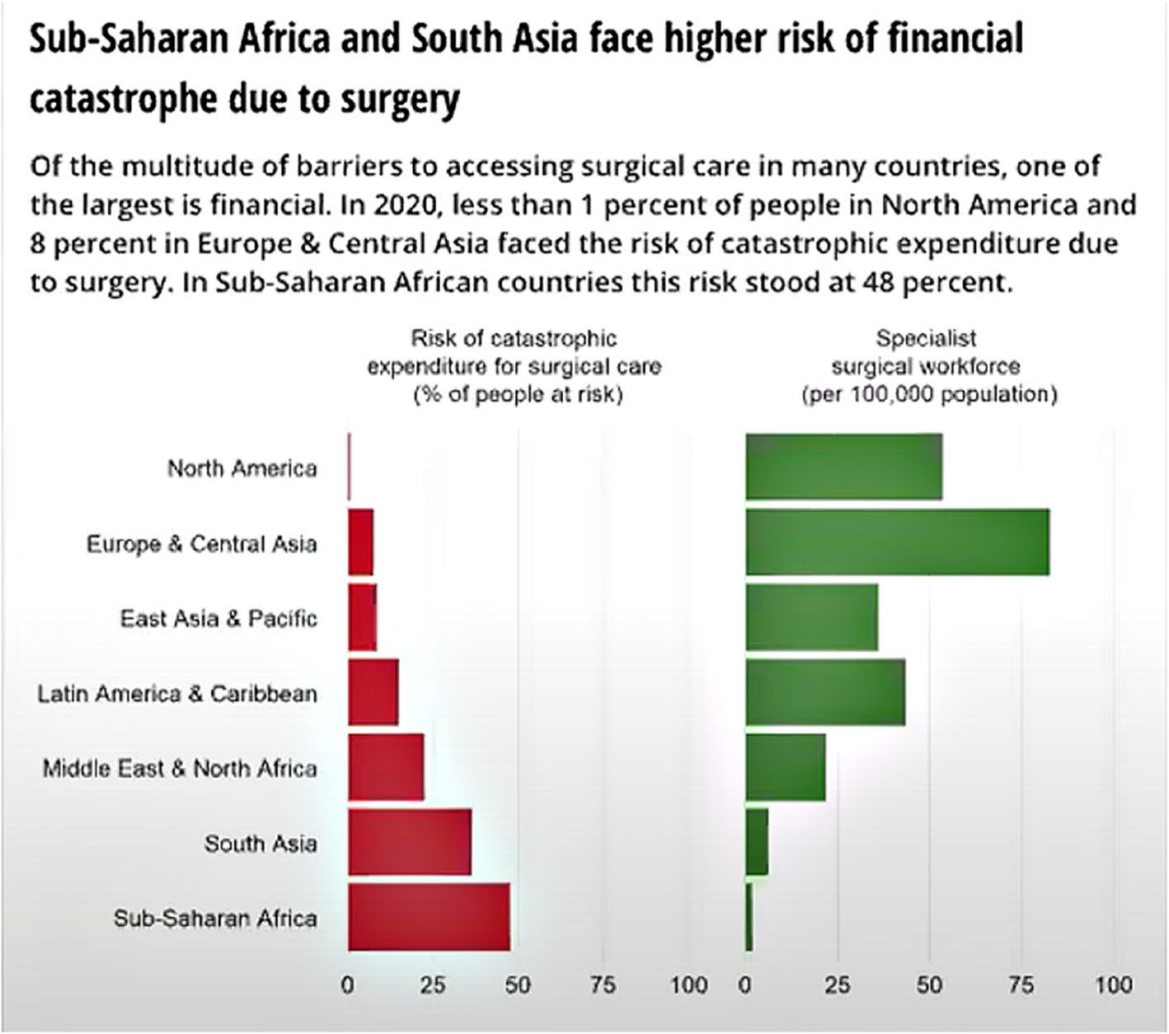
Sub-Saharan Africa and South Asia face higher risk of financial catastrophe due to surgery [8]

Meara noted that these indicators are conceptually straightforward, but they can be difficult to collect. Nonetheless, data serve an important function in communication with the broader health community. For example, The World Bank’s 2020 Atlas of Sustainable Development Goals [7] juxtaposed two segments of data: the risk of catastrophic expenditure for surgical care and the specialist surgical workforce. This has enabled further analyses pertaining to countries’ relative risk of financial catastrophe, for example, as illustrated in Fig. 1. The inclusion of surgical data in The World Bank’s interactive data compilation is a relatively new development, he noted.

Meara played a video address from Dr Tedros Adhanom Ghebreyesus, WHO Director-General, on the subject of global surgery. Ghebreyesus described SOA care as essential for every health system. To achieve UHC, a country must offer access to safe, timely, and affordable surgical services. Nine of the 13 targets included in Sustainable Development Goal (SDG) 3 can only be achieved with improved surgical care. However, 5 billion individuals—including 90% of people living in poverty—lack access to quality SOA services. Investment is therefore vitally important, and WHO has estimated that global investments equaling US$350 billion will be needed between 2016 and 2030 in order to build appropriate SOA systems. Failure to deliver this investment could result in losses of US$12 trillion or more by the year 2030. To promote the transformation of global SOA systems, WHO will engage in a strategic policy dialogue with countries to identify systems gaps and to ensure that all countries invest in SOA as they work toward realizing UHC.

**Fig. 2:**
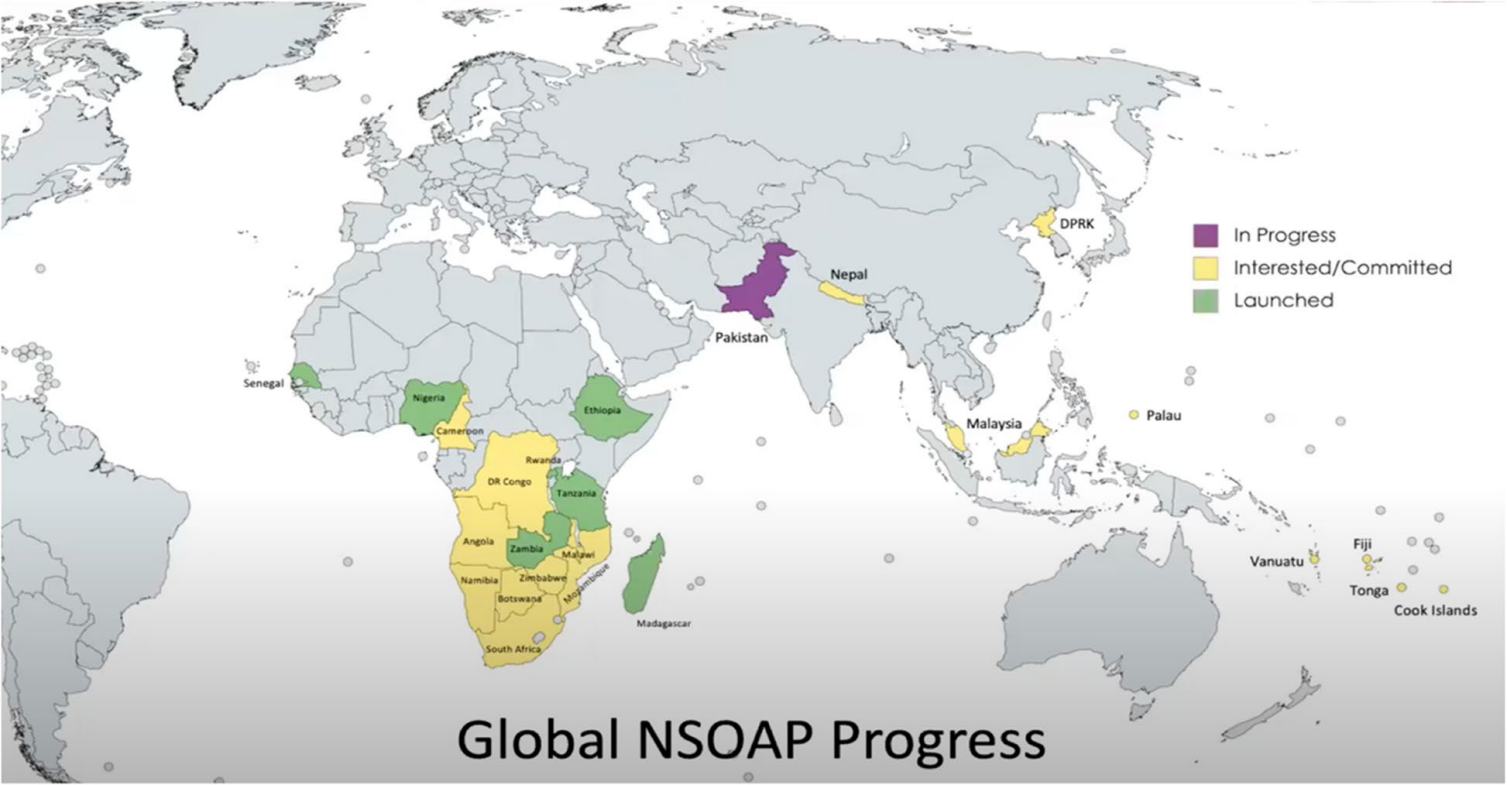
Global NSOAP progress (as of October 2021) [8]

Support from leaders such as Ghebreyesus has bolstered the LCoGS’s fourth output—the call for national surgical strategic planning [9]—with over 20 countries currently involved in this endeavor, said Meara. Figure 2 depicts the geographic distribution of countries engaging in the NSOAP process as of October 2021. The LCoGS outlined the domains that an NSOAP should address:

1. Workforce
2. Service delivery
3. Infrastructure
4. Information management
5. Finance
6. Governance

In 2020, the Western Pacific region of WHO passed Resolution WPR/RC71.R2 [10] endorsing the Action Framework for Safe and Affordable Surgery in the Western Pacific Region. This framework analogous to an NSOAP, and the resolution has moved the national planning process forward throughout this region.

### Recent funding for surgical capacity and national planning

Meara explained that the US Agency for International Development (USAID) can only spend funds as stipulated by congressional mandate in the form of a Department of State, Foreign Operations, and Related Programs (SFOPS) bill. For the first time, the 2022 SFOPS bill included specific language around surgical expenditure and national surgical planning (see Table 2). Meara emphasized the bill’s stipulation that within 90 days of the enactment of the act, the USAID administrator must brief the committees on appropriations on the planned use of funds for fiscal year 2022, representing a dramatic shift in USAID funding.

**Table 2:** USAID spending appropriated for strengthening surgical capacity [11]

“The USAID Administrator shall support efforts to strengthen surgical health capacity to address such health
issues as cleft lip and cleft palate, club foot, cataracts, hernias, fistulas, and untreated traumatic injuries in
underserved areas in developing countries. Strengthening surgical health systems includes the training of local
surgical teams to provide safe, sustainable, and timely surgical care, and assisting ministries of health to develop
and implement national surgical, obstetric, trauma, and anesthesia plans.”

Meara concluded by highlighting four conceptual aspects of global surgery. First, it must be integrated into national health priorities and must not be treated as a vertical intervention. The entire surgical ecosystem should be part of national health prioritization. Second, global surgical care delivery can aid in the achievement of UHC and the SDGs. Third, a change in USAID financing and funding appears to be taking place, and this development may influence other funders. Fourth, the COVID-19 pandemic has demonstrated that global surgery capacity and surgical workforce play beneficial roles in a pandemic response. Park remarked that investment in surgical capacity brings value to practically all aspects of health systems.

## The role of surgical care in ending cervical cancer

Dr Cherian Varghese, cross cutting lead, NCD and special initiatives, of the Department of Noncommunicable Diseases at WHO, Switzerland spoke in place of Dr Nothemba Simelela, WHO Assistant Director-General, to highlight the cervical cancer elimination initiative. He described cervical cancer as one of the most inequitable diseases in the world, and global inequities in prevention, screening, and treatment affect cervical cancer incidence and outcomes. For example, the death rates associated with cervical cancer are three times higher in low-income countries than in high-income countries [12]. In his experience treating cervical cancer, Varghese has witnessed patients facing extremely challenging situations, financial hardships, and social ostracization resulting from the disease. He described the current widespread inequity in access to treatment including surgical care as an injustice that manifests in cervical cancer.

### World Health Organization’s global strategy to eliminate cervical cancer

Varghese informed that the WHO Global Strategy to Accelerate the Elimination of Cervical Cancer as a Public Health Problem is a major step in advancing cervical cancer control [13]. This strategy recommends that by 2030:

- 90% of girls aged 9-14 should be vaccinated with the human papillomavirus vaccine
- 70% of women should be screened
- 90% of women diagnosed with cervical cancer and pre-cancer should be appropriately managed

This endeavor involves surgical capacity and requires surgeons, gynecologists, and obstetricians to work together on a care continuum. The integrated approach includes primary health care and screening, pre-cancer management, and the management of invasive cancers. Varghese suggested that cervical cancer can serve as a platform to raise awareness about inequity and increase urgently needed surgical capacity across the continuum of care for women, from pre-cancer management to palliative care. WHO is working through its surgical and maternal and childhood health programs to ensure that every woman in need of surgical support receives it.

## Service gaps in sexual and reproductive health

Dr Natalia Kanem, executive director at United Nations Population Fund (UNFPA), US, emphasized the shared commitment in achieving SDG 3 (ensuring healthy lives and promoting wellbeing for all at all ages) and SDG 5 (achieving gender equality and empowering all women and girls). UNFPA, which is the United Nations agency responsible for sexual and reproductive health, uses population data and evidence as the basis for all actions within the agency. These data are used to identify people affected by disparities and to explore the causes of these disparities.

### Preventable complications due to lack of access

In many parts of the world, women and girls—particularly those from marginalized populations—have the least access to health services. Furthermore, women and girls are often the hardest to reach with care, especially in poor and rural parts of the world. Each day, approximately 800 women die from complications from pregnancy and childbirth [14]. Adolescent girls have an elevated risk of dying from pregnancy-related complications. Access to sexual and reproductive health services has been further hindered by service disruptions and movement restrictions related to the COVID-19 pandemic. Fear of contracting the COVID-19 virus has caused some women to avoid seeking care. Unsafe abortions and childbirth complications have increased, leading to thousands of preventable maternal deaths. Kanem emphasized that the COVID-19 pandemic has exposed the growing inequities that were already present before the pandemic began.

### Geography-related tendencies toward over-medicalization or insufficient treatment

Kanem described what she considers to be an “inexcusable” paradox in maternal health outlined by Suellen Miller: the tendency to provide too much care too soon or too little care too late [15]. Normal pregnancies and births are routinely over medicalized, while high-risk mothers in the least developed countries receive little to no medical attention. Elective caesarean sections and the unnecessary use of non-evidence-based interventions have increased. Simultaneously, more than 800 women and girls die worldwide each day from pregnancy and childbirth complications that could be prevented with basic health services or the services of a surgeon or anesthesiologist. In the pursuit of UHC, equitable healthcare access for women and newborns is vital in meeting the needs of vulnerable populations. Kanem added that before pregnancy occurs, efforts are needed to protect adolescent girls, empower them with an understanding of their rights and choices, educate them about pregnancy and motherhood, and prevent girls from being coerced into pregnancy.

### United Nations Population Fund advocacy efforts

Given that over 5 billion people globally lack access to safe, timely, and affordable surgical care—resulting in millions of annual deaths—UNFPA works in over 120 LMICs to advocate for the inclusion of sexual and reproductive health services, said Kanem. Working with governments contending with limited budgets, the agency emphasizes the importance of considering the sexual and reproductive health needs of the population in determining which services to include in UHC or a national health plan. WHO’s 2019 monitoring report for global UHC indicates that reproductive, maternal, newborn, and child health service coverage was only slightly above 50% in low-income countries [16]. UNFPA strives to demonstrate the benefits of incorporating these services into national packages.

### Role of surgery in fistula prevention and treatment

Kanem stated that obstetric fistula is a prime indicator of the exclusion of impoverished women and girls from adequate health care. Fistulas of the uterus can be caused by prolonged, obstructed labor. In the absence of skilled medical care—including timely access to emergency caesarean section—fistula can occur. In many cases, this is accompanied by a stillbirth or death of the newborn within the first week of life. Women with fistula have a hole in the birth canal and sometimes in the rectum, and this can cause uncontrollable leaking of urine or feces. Thus, obstetric fistulas are stigmatizing and can lead to social exile from a community. Kanem recalled visiting women recovering from corrective surgery for fistula, which is often complex and difficult, and witnessing the return of a sense of dignity and belonging. However, in many low-income countries, financing mechanisms for fistula treatment are virtually non-existent.

### Campaign to End Fistula

Partnering with numerous organizations, UNFPA launched a global Campaign to End Fistula in 2003 [17]. The campaign is currently working around the world to increase awareness about protracted labor, drive action when fistula occurs, and provide funding mechanisms to enable access to surgery for the poorest women. Kanem emphasized that many fistulas could be prevented with increased access to appropriate emergency obstetric services for underserved populations and with increased attention to this issue from providers of primary care and specialized medical services. A balanced health system—that is, one that avoids providing too much care too soon in some countries and too little care too late in others—would provide safeguards for the wellbeing of all women and girls. Park added that maternal mortality ratios have increased in some LMICs during the COVID-19 pandemic, increasing the urgency of addressing issues related to pregnancy and childbirth.

## Expansion of UHC in Iran

Prof Dr Ali Jafarian, professor and former chancellor at Tehran University of Medical Sciences, Iran, discussed strategies Iran has utilized in the past 40 years to create educational pathways for surgical care providers and to scale up the surgical workforce. He outlined four pillars of UHC: (1) availability of services, (2) resources and equipment, (3) demand, and (4) access to health services.

### Availability of services, resources, and equipment

Approximately 30 years ago, Iran began addressing the first pillar of UHC—availability of services—by building its medical infrastructure, said Jafarian. The nation’s Ministry of Health and Medical Education, which is responsible for both community and university hospitals, expanded its university offerings to increase the number of medical schools, nursing schools, and midwifery schools, thereby expanding the nation’s capacity to produce graduates in these areas. The number of medical schools in Iran grew from 9 schools in 1979 to more than 70 schools in 2021. Moreover, enrollment continues to increase annually. Iran created a sixfold increase in its residency programs—which include SOA programs—in the past 40 years. The numbers of nursing and midwifery students have scaled to the point of a surplus of midwives and, in some provinces, nurses. The creation of a straight path for residencies 20 years ago has enabled more practitioners to be trained and has contributed to filling gaps in the country’s workforce of specialty providers. For example, medical students were able to begin their residencies in anaesthesia during their internships, resulting in an increase in the number of anesthesiologists.

### Demand and access to health care services

The demand for health care services in Iran currently remains high, noted Jafarian. In response to the need for more hospital facilities in small cities, the Iranian government and resource allocators constructed small hospitals with 64–96 beds around the country. However, maintaining these hospitals and equipping them with sufficient human resources, facilities, and equipment is hugely expensive due to the high levels of demand for services. He explained that the national parliament pushed for the creation of these small hospitals—despite the fact that serving patients in numerous small hospitals is less cost-effective than operating fewer large hospitals—to meet the public’s demand for better access to care in their communities. That being said, a referral system is in place for transferring some patients from facilities in small cities to other facilities in the centers of provinces and to tertiary care hospitals. Although Iran has a strong primary health care system that is well-established at the village level, access issues persist. In the past, approximately 70% of the nation’s population lived in rural settings; in 2021, that figure had dropped to 30%. With 70% of the population now living in large cities and their suburbs, primary health care planning must adjust to this population change. The Iranian government is currently developing needed changes to make primary health care more accessible given the country’s current demographics and geographical distribution of the population, he added.

### Surgical response to the COVID-19 pandemic

Jafarian remarked that as the COVID-19 pandemic unfolded worldwide, Iran’s surgeons quickly created new plans for the provision of emergency and essential surgeries. For instance, the Hepatobiliary Surgery and Liver Transplantation Division of the Imam Khomeini Hospital Complex—the largest hospital in Iran—developed a plan early in the pandemic to determine which patients would receive operations in order to decrease hospital usage and free up capacity for patients with COVID-19, who required 400–500 of the beds in the 1,100 bed hospital. After planning, the next step was to reallocate part of the surgical workforce to assist the physicians and specialists treating COVID-19 patients. Organ transplants continued without deferment during the pandemic, given the short window of time in which organs remain viable for transplant. Anesthetists were in great demand to cover the needs of COVID-19 patients in the intensive care unit, particularly at the beginning of the pandemic. Jafarian emphasized the need for more anaesthesia specialists throughout Iran and in its large hospitals.

### Workforce distribution

Iran has a mandatory service program in which all graduates from medical universities must serve in rural areas or in peripheral cities for 1–8 years, depending on the residency program, Jafarian explained. This service program has led to fewer referrals to other countries and has decreased fetal and maternal mortality rates to the SDG levels. These achievements are accompanied with the challenge of retaining young practitioners in peripheral areas of the country to maintain an adequate workforce. Additionally, the overproduction of professionals in certain areas, such as midwifery, has led to workforce supply exceeding the demand, leaving some practitioners unable to find work. Park noted that in the face of the current global shortfall of almost 6 million nurses, Iran appears to be an outlier in terms of strategic planning to achieve adequate nursing and health care workforces [18].

## National initiatives to expand surgical care in Ethiopia

Dr Lia Tadesse Gebremedhin, Minister of Health at the Federal Ministry of Health (MOH), Ethiopia, discussed Ethiopia’s scale up of surgical care services and the effect of the COVID-19 pandemic on surgical care delivery in the country. As part of the nation’s commitment to achieving UHC, Ethiopia has prioritized essential and emergency surgical interventions in revising its social service package.

### Surgery prioritization during the COVID-19 pandemic

Ethiopia’s COVID-19 mitigation efforts included extensive public health and social measures during the first months of the pandemic, which presented challenges in providing essential services, including surgery. Among these challenges were the need to prepare facilities to serve COVID-19 patients, uncertainties around how best to manage and provide essential services in the midst of a pandemic, and fear within the community around the risk of exposure to the virus when accessing care. In the early months of the pandemic, Ethiopia established a COVID-19 response task force to ensure the continuity of essential services and prioritize surgical services, particularly emergency surgery. Interventions included the rapid testing for all surgical patients and, once they became available, the provision of vaccines for health workers and communities. By late 2021, Ethiopia had successfully returned to pre-COVID-19 statistics and has even improved in some areas in comparison to before the pandemic.

### Saving Lives Through Safe Surgery Initiative 

The Saving Lives Through Safe Surgery (SaLTS) initiative was implemented in Ethiopia between 2016 and 2020 [19, 20]. Tadesse Gebremedhin stated that Ethiopia has recently revised its next five-year national SOA care strategy in collaboration with regional health bureaus and partners, notably associations of health professionals aiming to improve equitable access to high quality and safe essential and emergency surgical and anaesthesia care. The initial SaLTS strategy involved the establishment of a structure implemented within the MOH, regional bureaus, and SaLTS committees in health facilities.

#### Building workforce and surgery infrastructure

The SaLTS initiative increased budget allocations to improve access to surgery at various intervention levels. This enabled expansion of surgical workforce development for both pre-service and in-service, resulting in significantly increased surgical volume. Infrastructure is a key priority, and the MOH has focused on infrastructure for enhancing surgical services in primary hospitals and in primary health care centers. Tadesse Gebremedhin emphasized that the gaps in access to safe surgery cannot be met by relying solely on general hospitals. Targeting growth in primary care centers, Ethiopia has constructed 400 health centers in the past three years. Due to the challenges in equipping and staffing these centers, only about 10% of the newly constructed facilities are operational as of late 2021. A focus area for 2022 is to secure the necessary equipment and workforce to operationalize all facilities. The MOH is also working to increase oxygen capacity, a key need for surgical services. The demand for oxygen capacity has been compounded by the COVID-19 pandemic, and Ethiopia has capitalized on this opportunity to expand its oxygen capacity nationwide. Through public and private sector engagement, Ethiopia has tripled its national oxygen capacity since the start of the pandemic in 2020.

#### Surgical care indicator evaluation

Tadesse Gebremedhin explained that Ethiopia has incorporated key surgical care indicators into its monitoring and evaluation framework for SOA care. Examples of key indicators include surgical volume, surgical site infection rates, adverse outcomes from anaesthesia, and perioperative mortality. These indicators are tracked and monitored regularly, and qualitative safety improvement projects have been designed and executed. A surgical safety checklist was implemented nationally, resulting in the reduction of perioperative mortality and surgical side effects.

#### Service provider training capacity

Ethiopia is working to expand the health provider workforce and has developed a 2020–2030 plan for training medical specialists and mid-level health care providers. This national plan provides a framework for investment from partners and the government, and it anchors the newly developed, rigorous standards to existing training programs. Tadesse Gebremedhin highlighted the simultaneous goals of expanding training services and improving the quality of existing training programs. Quality improvement involves equipping training facilities with simulation labs and other technologies, as well as needed faculty and an enabling environment. Ethiopia is focusing efforts to train skilled surgeons in 12 major teaching hospitals. These practitioners will then immediately contribute to meeting the service gap of 5 million annual surgeries and reduce wait times for surgery, because long wait times can compromise surgical outcomes. Expanding surgical training capacity requires investment in infrastructure, and creating three new training institutions in Ethiopia will cost approximately US$2 million. The nation is working to mobilize resources to generate needed investment.

Additionally, Ethiopia is working to increase the workforce of highly competent obstetrician-gynecologists and midwives, said Tadesse Gebremedhin. Maternal mortality remains a key challenge requiring nationwide improvement in surgical interventions and caesarean rates. Currently, the country’s caesarean rate is low, at approximately 4%. In 2021, Ethiopia opened an anesthesiology postgraduate program, and the long-term maintenance of this initiative will necessitate additional investments in faculty training. Another focus area is training for emergency, critical care, and injury services. Ethiopia’s strategy also incorporates community interventions for early detection, including pre-hospital services and toll-free telephone hotlines. The MOH is also working to engage university hospitals and link them with city administrations to strengthen emergency response and improve timely emergency surgical interventions. The COVID-19 pandemic has created many challenges, and Ethiopia is striving to use these difficult circumstances as an opportunity to make progress toward achieving UHC. Park noted that while surgeons generally focus on curative services, efforts to prevent injuries might reduce the service burden.

## Discussion

### Achieving SDG UHC targets by 2030

Park and Uribe-Leitz moderated the panel discussion that followed the framing talks. Uribe-Leitz remarked that the 2030 target date for the SDGs is only 9 years away, and the current COVID-19 pandemic has created monumental challenges in meeting health needs. He asked about changes needed to achieve UHC in terms of SOA care by 2030.

#### National surgical strategic planning

Meara remarked that the tremendous success in Ethiopia can serve as a guide for other nations. Ethiopia was one of the first countries to develop an NSOAP as part of the SaLTS initiative. This program has accomplished a remarkable number of improvements in a relatively short period of time and has shown the world that it is feasible to achieve such substantial change. In 2018, the Southern African Development Community endorsed a resolution to prioritize surgical care [21]. The Pacific Islands have also shown strong leadership in national surgical strategic planning, with WHO’s Western Pacific Regional Office passing resolution WPR/RC71.92, Safe and Affordable Surgery, in October 2020 [10]. He noted that these changes have all occurred since 2015, demonstrating that nations and regions can initiate dramatic shifts in a relatively short period of time.

#### Standards, capacity building, and public-private engagement

Varghese commended Iran and Ethiopia for the substantial improvements they have achieved in his specialty area of noncommunicable diseases (NCD). The SDGs set ambitious targets such as SDG 3.4, which is the target of reducing premature mortality from NCDs by one third. The indicators for this target include the mortality rate attributed to cardiovascular disease, cancer, diabetes, and chronic respiratory disease—four conditions for which surgical interventions are critical. He emphasized that during the development of national UHC benefits packages, surgical interventions should be included under the minimum universal coverage benefits. In India, public health standards have been established for district hospitals that outline (1) which surgical interventions should be available, (2) which practitioners should perform them, and (3) the infrastructure requirements for those services. The formalization of these practices with written standards and compliance monitoring can foster scaling of surgical services.

Short-term capacity building must also be considered in expanding UHC, said Varghese. WHO is working with the United Nations Institute for Training and Research to expand surgical capacity for surgical oncology. Efforts have already taken place in Zambia, and these will extend to Rwanda and other countries. Areas in which women and men are dying from acute surgical intervention needs do not have the luxury of lengthy 8–10 year surgical specialty training programs. To address this gap, new methods of digital delivery and remote support can be used to decentralize some training services. Delays in care must also be addressed, because they can compromise patient outcomes. For example, in most parts of the world, a woman with a lump in her breast will not receive definitive treatment for 9–12 months. Service gaps and delays often disrupt the timeline for biopsy, pathology, mastectomy, and radiation. When system inefficiency is at play, a tumor that has a 90% cure rate with timely intervention might double in size and become stage 3, resulting in the survival probability decreasing to 10%. Therefore, service delays need to remain a focus in planning for the next decade. Anesthetic capacity is another consideration. Varghese noted that some locations are providing anesthetic training to paraprofessionals that is limited to specific procedures in order to deploy them into the field more quickly.

Varghese stated that in addition to capacity building, countries should ensure that financial protections cover surgical interventions and that appropriate public–private sector engagement is taking place. In many LMICs without standardized national insurance programs, the private sector is the primary provider for the complicated procedures often needed to treat NCDs. Overarching programs are needed to create seamless service between public and private health care, especially in regard to surgical capacity and pathology capacity. Collective action is needed to make substantial change in a short period of time, and services should first be directed toward populations that currently receive the least care. After the greatest unmet needs are addressed, efforts can expand toward other portions of the population. Fractioning minimal resources and capacity should be avoided by using an integrated approach. WHO is monitoring UHC service coverage, and the improvement in the universal coverage index is not optimal. Scaling up is thus needed to achieve SDG targets.

#### Obstetric and newborn care, investment in training, stakeholder involvement, and innovation

Kanem remarked that the most important approach to meeting the 2030 SDG targets is to hold firm to the 2030 date. The COVID-19 pandemic has presented daunting challenges, but health care remains an established human right. The UHC mechanism must prioritize comprehensive emergency obstetric and newborn care services, including caesarean section. This effort involves surgeons, anesthetists, midwives, families, and the women in need of services, as well as government funding and investment from the private sector to sustain a well-functioning health system. These interventions have proven critical in assuring a woman and newborn safe passage through childbirth. Therefore, supporting the types of national and local surgical service networks discussed today is important.

Furthermore, the unbalanced geographic distribution of trained personnel contributes to inequitable health outcomes, particularly for nomadic communities and isolated villages, said Kanem. More investment in training is needed, and this should come at the community level. Moreover, this investment should support career development for nurses and midwives, who are the backbone of maternal health systems in LMICs. The SDGs serve as agreed-upon indicators for the assessment of access to quality services and care. Stakeholders in UHC efforts in the area of reproductive health are numerous and include women’s groups, youth groups, survivors of fistula and reproductive cancers, any victims of a dysfunctional health system, the private sector, and national parliaments. Given that parliament controls funding, devises legislation, and appropriates budgets, this body can work to remove barriers to UHC. Kanem suggested that advocates for SOA care should avoid technical jargon to help people outside the field of medicine understand the aims of UHC and translate global UHC commitments into concrete national action.

Technology and innovation can also play a role in expanding health care. For instance, supply chain management—which has seen significant challenges during the COVID-19 pandemic—can be optimized by developing alternative key obstetric products that do not require a cold chain. Recent examples of such advances include stable carbetocin and tranexamic acid. New solutions can be generated with innovation. Kanem reiterated that health care must be understood as a human right; the needs of surgeons, anesthetists, midwives, families, and women should be considered; and funding and backing then need to be put in place.

#### Fostering resiliency with redundancy

Jafarian drew an analogy between the military and the health care workforce. In many countries, decades pass in which the military service is not involved in war, yet the military is maintained such that it is available when needed. The COVID-19 pandemic has demonstrated the value of having health care capacity available when major health events occur. This capacity comes with redundancy during normal times; thus, a resilient system requires some redundancy. In health care, creating and maintaining some redundancy necessitates budget, infrastructure, and workforce. He remarked that after the COVID-19 pandemic, governments should understand the value of capacity and associated redundancy. Surgeons, obstetricians, and anesthetists should not be constantly working around the clock. Instead, some redundancy should be built in so that when major events take place, a highly trained workforce has the capacity available to adequately address them.

Iran sets the highest standards for practitioners performing SOA care, said Jafarian. These individuals should be specialists and board eligible in order to practice in these areas. However, this standard is expensive given the years of training it requires. These three specialties require individuals to pass 7 years of medical school followed by 4 years in residency, constituting 11 years of training before they are able to practice independently. Therefore, maintaining the highest standards is an expensive endeavor.

#### Improving rural access with decentralization, investment, and training

Tadesse Gebremedhin stated that the current COVID-19 pandemic highlights the importance of creating resilient, sustainable programs. She emphasized the need to invest in primary health care systems. In a country such as Ethiopia, much of the population lives in rural areas and cannot access larger hospitals. Thus, ensuring that these communities have access to safe surgery in primary care hospitals and centers is critical. Investment can enable the decentralization of key services for NCDs, injuries, and other essential surgical needs. This provides access to services for rural populations while also offering larger hospitals the opportunity to focus on more complicated services. Without this decentralization, cases that could be served at the primary care level are instead treated at larger hospitals, and this dynamic can compromise the quality of care that hospitals are able to offer. Building capacity within a decentralized system creates resiliency to meet challenges such as the COVID-19 pandemic, she added. Moreover, financing and resource allocation heavily influence the strength of a system, and investment from governments, donors, and the private sector is critical to improving surgical care. Lastly, a highly trained workforce requires sustainable expansion and quality improvement of training programs. This can be carried out through twinning programs with other institutions, but building strong, sustainable, internal training capacity cannot be dependent on external support. Focus on these areas can contribute toward solid progress on achieving the SDGs by 2030.

### Pediatric surgical care

Emma Bryce, a workshop participant from KidsOR, asked about the importance of including specialist-provided pediatric surgical care and pediatric and neonatal surgical equipment in wider surgical care plans. Kanem replied that in considering the impact that surgical correction or amelioration can have on the life course of a person with a disability, proper planning for pediatric needs is important. Furthermore, given the rate of car crashes in which young people are injured, awareness about public health measures such as seat belts and speed limits is needed.

### Increasing access with training alternatives, decentralization, and technology

Noting the time and expense required to obtain high-quality surgical certification, Kanem asked about experiences with para-surgical personnel, particularly for caesarean section. Park invited Tadesse Gebremedhin to discuss Ethiopia’s midwifery model and surgical officer system. She replied that during Ghebreyesus’s tenure as the country’s Minister of Health, Ethiopia created an Integrated Emergency Surgery program to produce emergency surgical officers. The program is offered to health officers—who hold four-year bachelor’s degrees in public health—and provides three years of master’s-level training on emergency surgeries, including caesarean sections. Many of Ethiopia’s primary hospitals are staffed with emergency surgical officers performing life-saving surgeries. These professionals will also be utilized in the newly constructed health centers once they become operational. The creation of emergency surgical officers has proven to be a strong support in improving access to surgery.

More recently, Ethiopia has created a clinical midwifery master’s program in which midwives are trained to perform caesarean sections. A current challenge to this strong program is the robust expansion of medical schools and physician training that Ethiopia has undertaken in recent years. In fact, due to the large numbers of graduating doctors and resulting job shortages, the country is working to expand residency training programs. In this process, mid-level training for para-surgical officers is under discussion. Additionally, para-surgical officers have raised concerns regarding career path opportunities. Tadesse Gebremedhin stated that while challenges are at play, this programming has had a tremendous effect on access to safe surgeries.

Jafarian noted that decisions regarding para-surgical professionals should be context-based, because countries have differing needs. While Ethiopia has experienced a shortage of medical school graduates and residents, Iran has sufficient numbers of graduates. Furthermore, high standards are firmly established in Iran, and lowering these standards could be problematic. These issues are complex, and each country must find the solution that best fits its specific needs.

Varghese remarked that too often, the concentration of services to a specific category of providers has hampered the attainment of high levels of health coverage. Decentralizing care—while also putting some supportive supervision in place—can increase capacity. During the COVID-19 pandemic, decentralization served as an adaptation mechanism that immediately improved service coverage in many parts of the world. Likewise, decentralization for surgical care is feasible. The establishment of district surgical teams, which may include mobile and support operations, can expand the provision of coverage to various areas within the district. The suffering caused by the COVID-19 pandemic across the world serves as a wakeup call for needed change, he added. Decentralized care, stepdown care, and self-care supported by digital technology are additional approaches to expanding access to care. Health providers waited for two decades for guidance on teleconsultation, yet once the COVID-19 pandemic struck, governmental guidance was issued in a matter of only three weeks. This momentum can be harnessed to decentralize and socialize medical services with an empowered group of recipients and to support these services with adequate regulatory mechanisms. A joint commission from *The Lancet* and the *Financial Times*recently published a report indicating that digital health is maximized with global governance [22]. Varghese stated that new technology for cervical cancer screening utilizes artificial intelligence to interpret cytology and visual inspection. Such innovations can reduce provider-related delays in treating cancer and other similar conditions, which in turn could positively affect outcomes.

### Pregnancy complication prevention in low-income settings

A workshop participant from the German Medical Student Association asked about screening tools that can be used in low-income settings to identify women at the highest risk for developing birth complications. Early identification could enable these women to be transported to hospitals to avoid emergency caesarean section, an intervention that can be difficult to provide in rural settings. Tadesse Gebremedhin replied that detection of high-risk maternal cases takes place at different levels. Antenatal care is the key modality for screening pregnant women. Given that the majority of Ethiopia’s population is in the rural sector, community health extension workers are utilized to provide prevention and health promotion services in people’s homes and in community-level clinics referred to as “health posts.” Pregnant women can receive antenatal care at health posts and at primary care centers, with laboratory tests provided at the latter. Ethiopia is also working to improve access to ultrasound screening across primary care units, as this access is currently limited. Although not all pregnant women opt to visit the clinics, the rates of women receiving antenatal care are high in the country. In accordance with WHO guidance [23], Ethiopia strives to ensure that pregnant women receive four antenatal care visits, with at least one of these taking place at a primary health care center. Women with identified risk are referred to general hospitals that provide services across the continuum of care.

Kanem emphasized the importance of training in meeting the WHO standards. UNFPA works with countries around the world to carry out the work Tadesse Gebremedhin described. Training is directly related to quality of care and should be provided as appropriate for a country’s specific context. WHO and UNFPA support the Network for Improving Quality of Care for Maternal, Newborn and Child Health, which works toward the goal of reducing the number of maternal and newborn deaths and stillbirths in health facilities by 50% [24].

### Addressing global inequities in service distribution

Uribe-Leitz asked how the uneven distribution of surgical services can be addressed to avoid the concentration of global surgery in certain regions and lack of access in others. Meara replied that the distribution of services is related to funding. The SFOPS appropriation bill creates a new funding stream, and equity and justice should be factors in determining how those USAID funds are spent. Given that the USAID administrator has 90 days in which to determine spending vis-à-vis the SFOPS bill before briefing the appropriations committee on the planned use of funds, Meara highlighted the opportunity that has emerged in the current moment. He suggested that MOHs contact USAID regarding this congressional mandate. He encouraged organizations such as USAID, The World Bank, and the Bill and Melinda Gates Foundation to invest in global surgery to a broader extent as part of national health planning priorities.

### Status of global surgery and closing remarks

Naomi Lee, senior executive editor at *The Lancet*, noted the remarkable examples of advances in global surgery presented at this workshop. She asked whether the status of global surgery as “the neglected stepchild of global health” has changed in the present day. Meara highlighted the high caliber of the panel, comprised of a chancellor emeritus, a minister of health, a leader from the WHO, and the executive director of UNFPA. The attention of high-ranking individuals to the issue of global surgery constitutes a shift from only 5 years ago. The current level of commitment to this issue indicates that global surgery no longer has the neglected status it once did. The developments discussed during the webinar—such as the remarkable scaling that Iran has achieved, the advances made through Ethiopia’s SaLTS initiative, the educational goals toward which WHO is working—demonstrate a renewed focus on a global surgical workforce ecosystem.

Tadesse Gebremedhin noted that advancing improved access and quality for essential surgical services depends upon many components, including infrastructure, training, advocacy, and financing. In addition, strong leadership is needed to further this agenda at the facilities level as well as at the national and global levels. Thus, a commitment to strong leadership is core to advancing the agenda. Furthermore, increased funding is needed. While national initiatives and strategies have brought substantial change in recent years, global surgery remains underfunded. Governments and donors can strengthen national systems, particularly with respect to training. Once a trained workforce is established, resources are required to adequately equip these providers. She called on donors and partners to support financing efforts for this crosscutting agenda that impacts numerous health indicators.

Varghese stated that surgery is “the great unifier.” Whereas multiple siloed areas can be found in the global public health agenda, global surgery is relevant to all large-scale health initiatives, to every major disease, and throughout the life course. Although surgery is no longer neglected in the way it once was, the need for international support remains. Domestic policy and financing are required to expand and sustain surgical training and surgical services capacity. Substantial lack of coverage for NCDs persists in rural areas. The concentration of high-end surgical capacity in urban areas needs to be redistributed; Iran and other countries have demonstrated that this is possible. He added that a WHO Academy is being established in Lyon, France as part of the organization’s global capacity-building programs [25]. The academy will feature simulation-based training programs and certified training programs. WHO designated 2021 as the “Year of Health and Care Workers” to highlight the role of surgeons, nurses, and other health professionals, and to acknowledge both their efforts and the heavy toll the COVID-19 pandemic has taken on the field [26].

Jafarian remarked that the importance of global surgery as a global health issue has only been acknowledged relatively recently, and that the initiative to advance global surgery is gaining momentum within the World Health Summit. He added that the 2022 World Health Summit will be co-hosted with WHO, offering an opportunity to further disseminate the topic of global surgery with the global health community.

Kanem recalled that when Allan Rosenfield, a prominent public health advocate and her former teacher, coined the acronym EMOC for emergency obstetric and newborn care, it was slow to gain traction. However, EMOC is now common practice. The field of SOA care has seen progress, but the need for strong advocacy remains. Birth should be safe everywhere, even in fragile contexts and settings in which preventable maternal deaths currently occur. SOA services should be incorporated into UHC as essential care. Kanem added that not only do these services save lives, they also save money. Rehabilitation services are also needed, particularly in pediatrics as well as for older patients accessing care. UNFPA advocates for universal access to sexual and reproductive health, and partnerships are important in improving and expanding these services. She commended the work of service providers, highlighting their ongoing efforts despite the challenges of the COVID-19 pandemic. Through their efforts, babies are safely delivered to happy mothers, and advocacy efforts strive toward realizing the goal of healthy births in delivery rooms around the world.

Park concluded the workshop by noting that Dr Atul Gawande, an endocrine surgeon, has been nominated to lead global health development at USAID. He emphasized that given the current alignment of funders, there has never been a better time to invest in the strengthening of surgical systems the world over.

## About the supplement

About this supplement This article has been published as part of BMC Proceedings Volume 17 Supplement 6, 2023: Universal Health Coverage: A Commitment to Essential Surgical, Obstetric, and Anesthesia Care, World Health Summit 2021 (PD 20). The full contents of the supplement are available online at https://bmcproc.biomedcentral.com/articles/supplements/volume-17-supplement-6.

## Data Availability

The dataset(s) supporting the conclusions of this article is(are) included within the article.

## Abbreviations

LMIC: Low- and middle-income country
MOH: Ministry of health
NCD: Noncommunicable disease
NSOAP: National surgical, obstetric, and anaesthesia plan
SaLTS: Saving Lives Through Safe Surgery
SDG: Sustainable Development Goal
SFOPS: Department of State, Foreign Operations, and Related Programs
SOA: Surgical, obstetric, and anaesthesia
UHC: Universal health coverage
UNFPA: United Nations Population Fund
USAID: US Agency for International Development
WHO: World Health Organization
